# Supplementary material for: Dual-function enzyme acts as a global c-di-GMP sink and local anti sigma factor antagonist to drive cellular differentiation
Source: PLoS Genet. 2026 Jun 3;22(6):e1012161. doi: 10.1371/journal.pgen.1012161 (PMC13232838; doi:10.1371/journal.pgen.1012161)
Supplement: S1 Fig — S. venezuelae rmdB mutant carrying either p3xFLAG-rmdB, p3xFLAG-rmdBΔGGDEF, p3xFLAG-rmdBAAA, or p3xFLAG-rmdBΔTM was grown in MYM complemented with hygromycin (50 µg/ml) at 30°C and 180 rpm. Samples were taken after 12, 16 and 20 hours of growth. 20 µg total protein were used for each sample. The anti-FLAG antibody (Sigma) was used for detection. (DOCX) [file pgen.1012161.s001.docx]

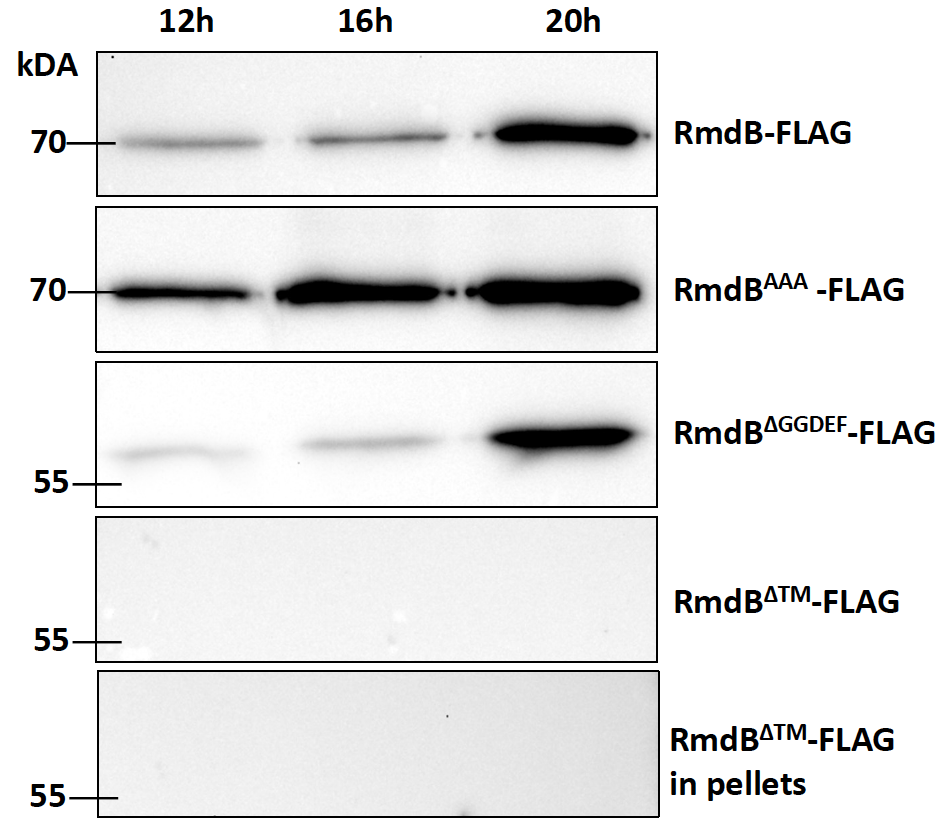


**S1 Fig. Western Blot analysis to study protein levels of different RmdB variants.** *S. venezuelae* *rmdB* mutant carrying either *p3xFLAG*-*rmdB*, *p3xFLAG*-*rmdB^ΔGGDEF^*, *p3xFLAG*-*rmdB^AAA^,* or *p3xFLAG*-*rmdB^ΔTM^* was grown in MYM complemented with hygromycin (50 µg/ml) at 30℃ and 180 rpm. Samples were taken after 12, 16 and 20 hours of growth. 20 µg total protein were used for each sample. The anti-FLAG antibody (Sigma) was used for detection.
