# Supplementary material for: Dual-function enzyme acts as a global c-di-GMP sink and local anti sigma factor antagonist to drive cellular differentiation
Source: PLoS Genet. 2026 Jun 3;22(6):e1012161. doi: 10.1371/journal.pgen.1012161 (PMC13232838; doi:10.1371/journal.pgen.1012161)
Supplement: S3 Fig — Cells were grown in MYM at 30°C and 180 rpm for 18 hours. FLAG-tagged proteins were immunoprecipitated using FLAG-tag-specific magnetic beads (Miltenyi Biotec). S. venezuelae ΔrmdB carrying the empty p3xFLAG plasmid served as a negative control. After Co-IP, eluates (=IP) and cell lysates (=Input) were analysed using western blotting (WB) and either the monoclonal anti-FLAG (Sigma) (C) or the polyclonal anti-WhiG (D) antibodies [33]. 20 µg total protein was used as input for each sample. (DOCX) [file pgen.1012161.s003.docx]

**
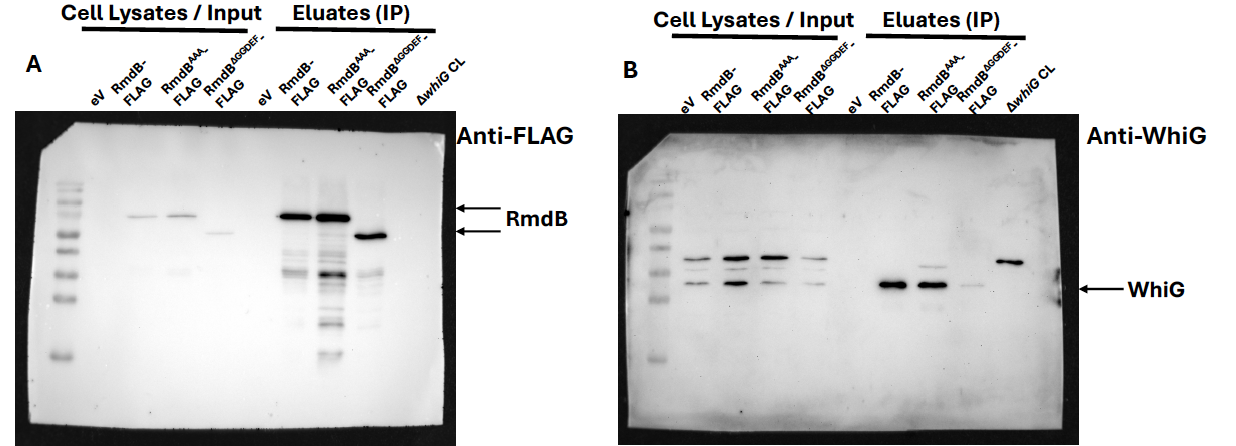
**

**S3 Fig. Co-immunoprecipitation (Co-IP) analysis using RmdB-FLAG, RmdB^AAA^-FLAG and RmdB^ΔGGDEF^-FLAG in *S. venezuelae* (non-processed images from Figure 3C (A) and Figure 3D (B)):** *rmdB-*FLAG, *rmdB^AAA^-*FLAG and *rmdB^ΔGGDEF^-*FLAG were expressed in the *S. venezuelae* *rmdB* mutant. Cells were grown in MYM at 30℃ and 180 rpm for 18 hours. FLAG-tagged proteins were immunoprecipitated using FLAG-tag-specific magnetic beads (Miltenyi Biotec). *S. venezuelae* Δ*rmdB* carrying the empty p3xFLAG plasmid served as a negative control. After Co-IP, eluates (=IP) and cell lysates (=Input) were analysed using western blotting (WB) and either the monoclonal anti-FLAG (Sigma) (C) or the polyclonal anti-WhiG (D) antibodies (Gallagher *et al.*, 2020). 20 µg total protein was used as input for each sample.
