# Supplementary material for: Dual-function enzyme acts as a global c-di-GMP sink and local anti sigma factor antagonist to drive cellular differentiation
Source: PLoS Genet. 2026 Jun 3;22(6):e1012161. doi: 10.1371/journal.pgen.1012161 (PMC13232838; doi:10.1371/journal.pgen.1012161)
Supplement: S4 Fig — Cells were grown in MYM at 30°C and 180 rpm for 18 hours. FLAG-tagged proteins were immunoprecipitated using FLAG-tag-specific magnetic beads (Miltenyi Biotec). S. venezuelae ΔrmdB carrying the empty p3xFLAG plasmid served as a negative control. After Co-IP, eluates (=IP) and cell lysates (=Input) were analysed using western blotting (WB) and the polyclonal anti-RsiG antibody [33]. 20 µg total protein were used as input for each sample. (DOCX) [file pgen.1012161.s004.docx]

**
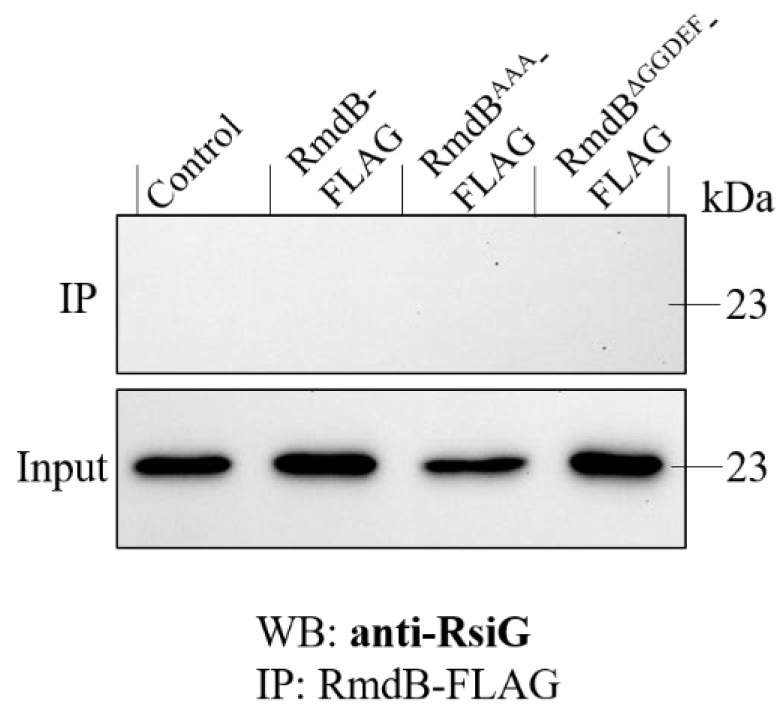
**

### **S4 Fig. Co-immunoprecipitation (Co-IP) analysis failed to detect interaction between RmdB and RsiG.** *rmdB-*FLAG, *rmdB^AAA^-*FLAG and *rmdB^ΔGGDEF^-*FLAG were expressed in the *S. venezuelae* *rmdB* mutant. Cells were grown in MYM at 30℃ and 180 rpm for 18 hours. FLAG-tagged proteins were immunoprecipitated using FLAG-tag-specific magnetic beads (Miltenyi Biotec). *S. venezuelae* Δ*rmdB* carrying the empty p3xFLAG plasmid served as a negative control. After Co-IP, eluates (=IP) and cell lysates (=Input) were analysed using western blotting (WB) and the polyclonal anti-RsiG antibody (Gallagher *et al.*, 2020). 20 µg total protein were used as input for each sample.
