# Supplementary material for: Dual-function enzyme acts as a global c-di-GMP sink and local anti sigma factor antagonist to drive cellular differentiation
Source: PLoS Genet. 2026 Jun 3;22(6):e1012161. doi: 10.1371/journal.pgen.1012161 (PMC13232838; doi:10.1371/journal.pgen.1012161)
Supplement: S5 Fig — GST-tagged rmdB was expressed from the pGEX.6p1 vector. His-whiG and tag less rsiG were expressed from pCOLADuet-1 from two separate multiple cloning sites [33]. Gene expression was induced with 250 µM IPTG and cells were incubated in LB at 16°C overnight. Purification of GST-RmdB, His-WhiG and RsiG was performed using the glutathione sepharose. The eluates were analysed via SDS-PAGE. (DOCX) [file pgen.1012161.s005.docx]

**
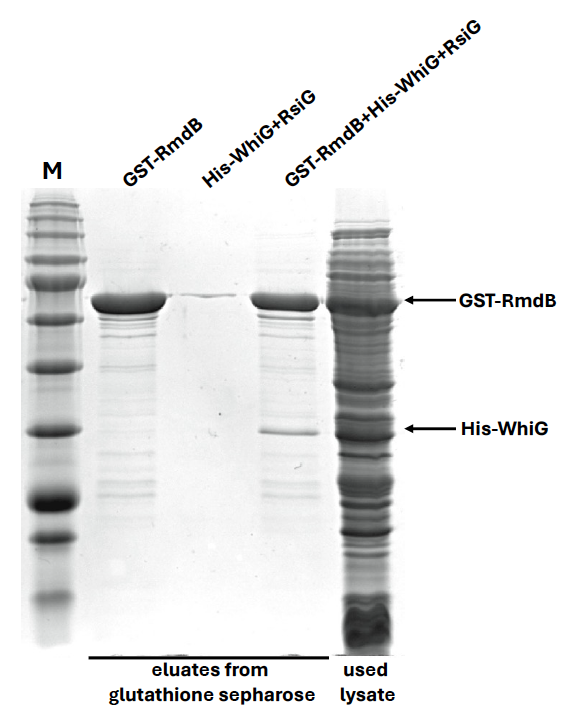
**

**S5 Fig. Co-purification of GST-RmdB and His- σ^WhiG^ from *E. coli* BL21 pLysS Rosetta.** GST-tagged *rmdB* was expressed from the pGEX.6p1 vector. His-*whiG* and tag-less *rsiG* were expressed from pCOLADuet-1 from two separate multiple cloning sites (Gallagher *et al.*, 2020). Gene expression was induced with 250 µM IPTG and cells were incubated in LB at 16℃ overnight. Purification of GST-RmdB, His-WhiG and RsiG was performed using the glutathione sepharose. The eluates were analysed via SDS-PAGE.
