# Supplementary material for: Dual-function enzyme acts as a global c-di-GMP sink and local anti sigma factor antagonist to drive cellular differentiation
Source: PLoS Genet. 2026 Jun 3;22(6):e1012161. doi: 10.1371/journal.pgen.1012161 (PMC13232838; doi:10.1371/journal.pgen.1012161)
Supplement: S6 Fig — GST-rmdBGGDEF was expressed with His-whiG (A) or alone (B) in E. coli BL21 pLysS from two separate plasmids (pIJ10914/His-WhiG and pET15b/GST-RmdBGGDEF). Gene expression was induced with 250 µM IPTG, and cells were incubated in LB at 16°C overnight. Purification of GST-GGDEFRmdB and His-WhiG was performed using the Ni-NTA matrix. Elution was performed using 3x washes with a 50 mM imidazole concentration and eluted with 250 mM imidazole. Eluates were analysed via SDS-PAGE. C. Uncropped gel image. Please note that the three prominent protein band in (A) were cut out from gel and analysed using MALDI-TOF to confirm identity of the relevant proteins. (DOCX) [file pgen.1012161.s006.docx]

**
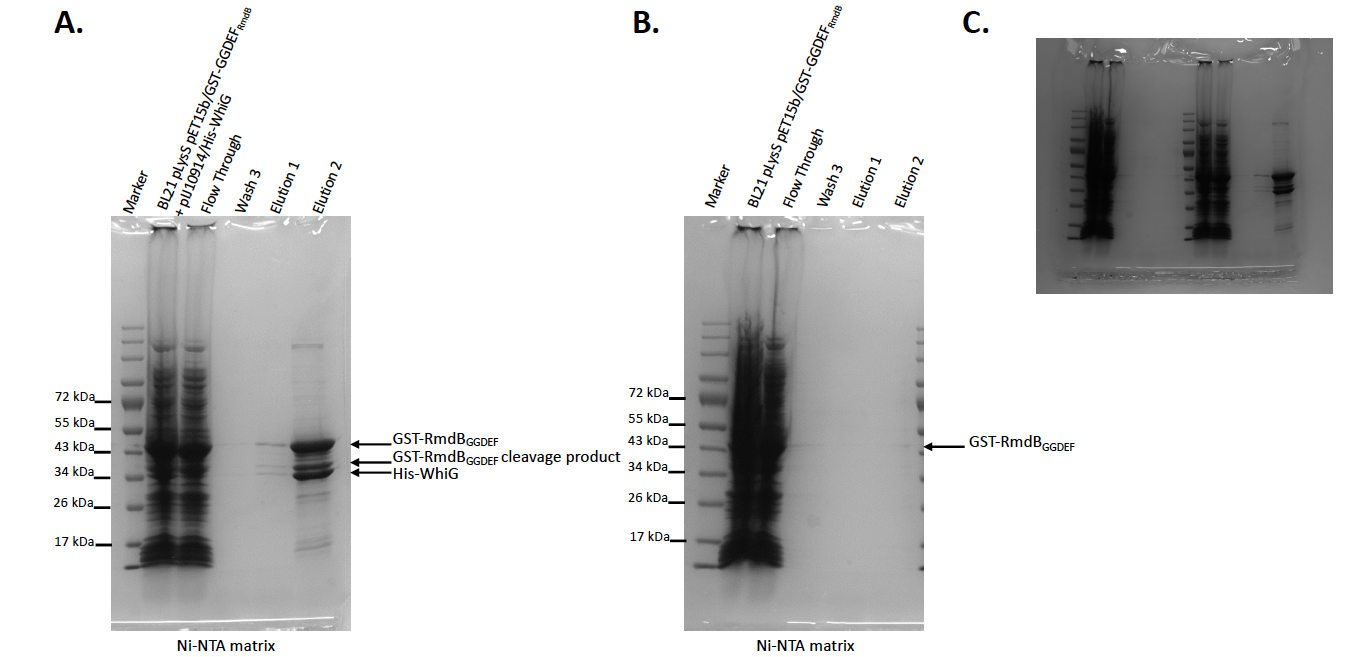
**

**S6 Fig. GST-tagged GGDEF domain from RmdB interacts with His-WhiG.** GST-*rmdB*GGDEF was expressed with His-*whiG* (A) or alone (B) in *E. coli* BL21 pLysS from two separate plasmids (pIJ10914/His-WhiG and pET15b/GST-RmdBGGDEF). Gene expression was induced with 250 µM IPTG, and cells were incubated in LB at 16°C overnight. Purification of GST-GGDEF_RmdB_ and His-WhiG was performed using the Ni-NTA matrix. Elution was performed using 3x washes with a 50 mM imidazole concentration and eluted with 250 mM imidazole. Eluates were analysed via SDS-PAGE. C. Uncropped gel image. Please note that the three prominent protein band in (A) were cut out from gel and analysed using MALDI-TOF to confirm identity of the relevant proteins.
