# Supplementary material for: Dual-function enzyme acts as a global c-di-GMP sink and local anti sigma factor antagonist to drive cellular differentiation
Source: PLoS Genet. 2026 Jun 3;22(6):e1012161. doi: 10.1371/journal.pgen.1012161 (PMC13232838; doi:10.1371/journal.pgen.1012161)
Supplement: S7 Fig — AlphaFold3 [29] was employed to model full-size RmdB and RmdBΔGGDEF (Δ258-421aa). The structural models are visualised using PyMOL. (DOCX) [file pgen.1012161.s007.docx]

**
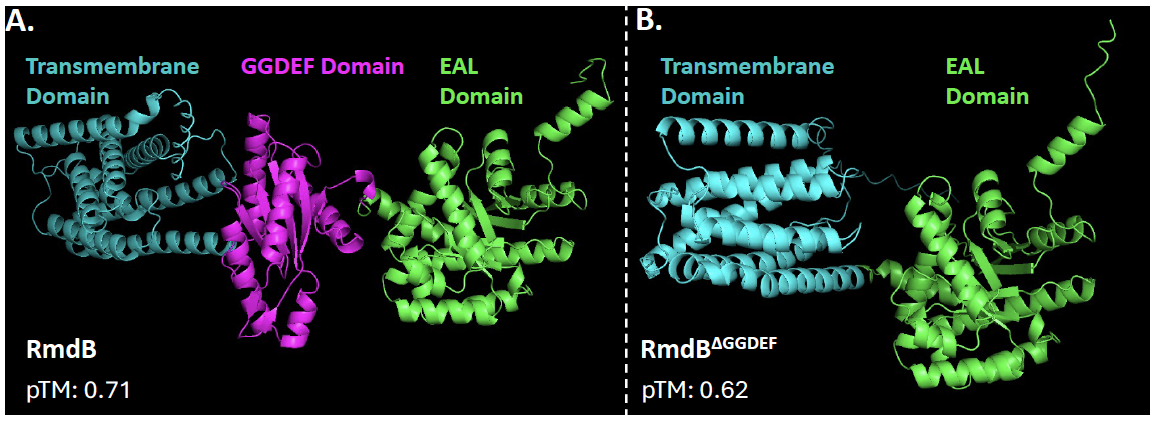
**

**S7 Fig. AlphaFold3 model comparisons of RmdB and RmdB^ΔGGDEF^.** AlphaFold3 (Abramson *et al.*, 2024) was employed to model full-size RmdB and RmdB^ΔGGDEF (Δ258-421aa)^. The structural models are visualised using PyMOL.
