# Supplementary material for: Dual-function enzyme acts as a global c-di-GMP sink and local anti sigma factor antagonist to drive cellular differentiation
Source: PLoS Genet. 2026 Jun 3;22(6):e1012161. doi: 10.1371/journal.pgen.1012161 (PMC13232838; doi:10.1371/journal.pgen.1012161)
Supplement: S8 Fig — Indicated genes were fused to either the T18 or T25 fragment of the adenylate cyclase from Bordetella pertussis on the pUT18/pUT18C or pKT25/pKNT25 vectors that were co-transformed into E. coli BTH101. Several clones carrying both plasmids were combined in 1 ml 1xPBS and 10 µl were spotted on LB agar supplemented with 5-Bromo-4chloro-3indolyl-ß-D-galactopyranoside (X-gal; 60 µg/ml), IPTG (0.5 mM) ampicillin (100 µg/ml) and kanamycin (50 µg/ml). Plates were incubated for either 40 hours at 30 °C (A, B) or for 56 hours at 25°C (C, D). Cytosolic fraction of RmdB (∆1–238aa) was used in BACTH assays as fusing either the T18 or T25 fragment to full size RmdB resulted in protein instability in E. coli. Original screening plates are shown on the left (A, C), with each colony carrying a number. Corresponding matrix explaining the identity of each spot is shown on the right (B, D). (DOCX) [file pgen.1012161.s008.docx]

**
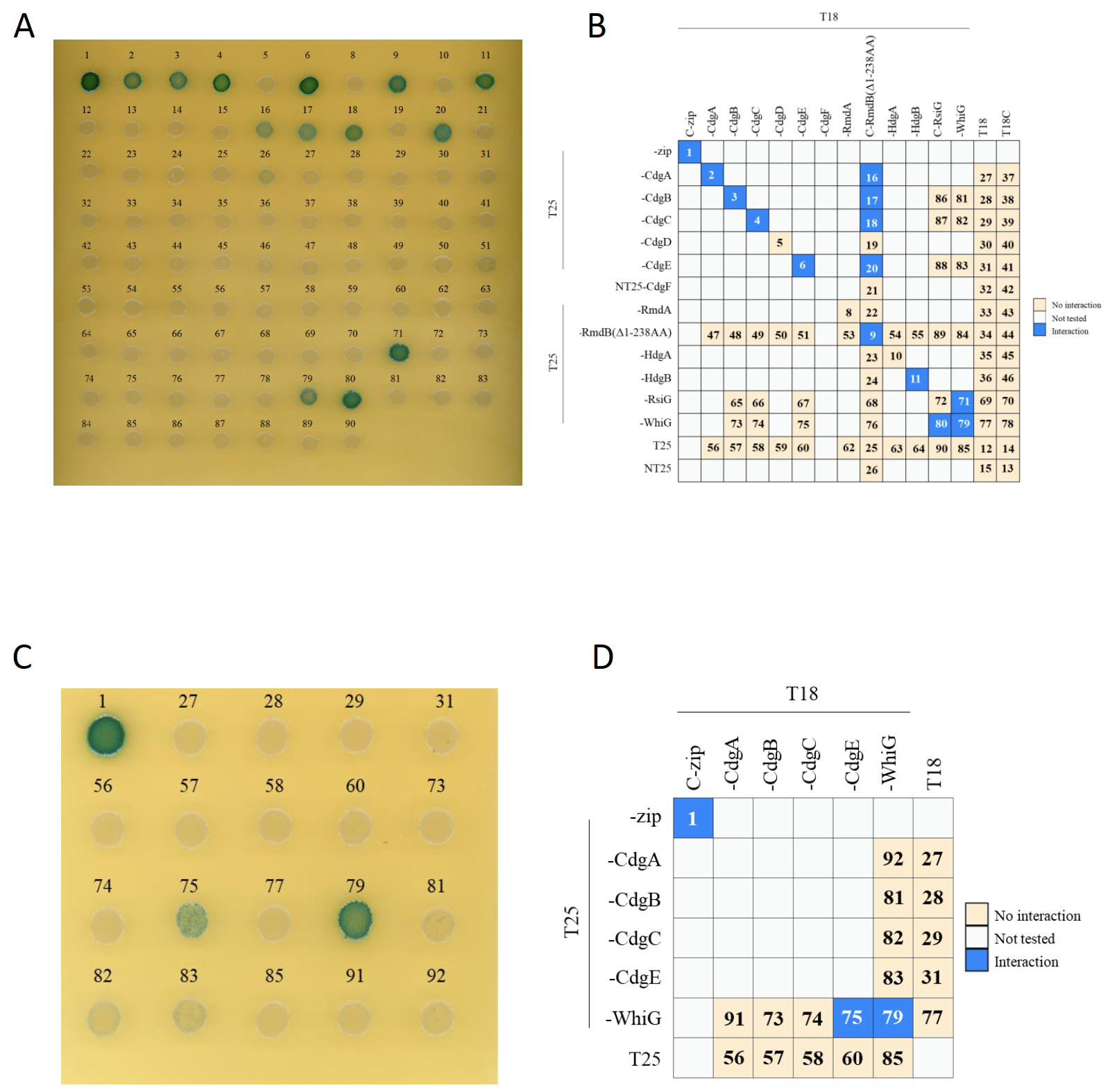
**

**S8 Fig. Bacterial Adenylate Cyclase Two-Hybrid (BACTH) assays reveal multiple interactions between RmdB and DGCs**. Indicated genes were fused to either the T18 or T25 fragment of the adenylate cyclase from *Bordetella pertussis* on the pUT18/pUT18C or pKT25/pKNT25 vectors that were co-transformed into *E. coli* BTH101. Several clones carrying both plasmids were combined in 1 ml 1xPBS and 10 µl were spotted on LB agar supplemented with 5-Bromo-4chloro-3indolyl-ß-D-galactopyranoside (X-gal; 60 µg/ml), IPTG (0.5 mM) ampicillin (100 µg/ml) and kanamycin (50 µg/ml). Plates were incubated for either 40 hours at 30 °C (A, B) or for 56 hours at 25°C (C, D). Cytosolic fraction of RmdB (∆1-238aa) was used in BACTH assays as fusing either the T18 or T25 fragment to full size RmdB resulted in protein instability in *E. coli*. Original screening plates are shown on the left (A, C), with each colony carrying a number. Corresponding matrix explaining the identity of each spot is shown on the right (B, D).
