# Supplementary material for: Dual-function enzyme acts as a global c-di-GMP sink and local anti sigma factor antagonist to drive cellular differentiation
Source: PLoS Genet. 2026 Jun 3;22(6):e1012161. doi: 10.1371/journal.pgen.1012161 (PMC13232838; doi:10.1371/journal.pgen.1012161)
Supplement: S1 Table — (DOCX) [file pgen.1012161.s009.docx]

**S1 Table. Strains, plasmids and oligonucleotides used in this study**

| **Strains / NT Strain collection number** | **Genotype or comments** | **Source or reference** |
| --- | --- | --- |
| ***S. venezuelae*** | | |
| NRRL B-65442 | Wild type | (NCBI Reference Sequence: NZ_CP018074.1) |
| SVNT26 | *rmdB*::*apr*, Apr^R^ | (Al-Bassam *et al.*, 2018) |
| SVNT39 | *rmdB*::*apr*; *attBΦBT1*::p3xFLAG-*rmdB*; Apr^R^, Hyg^R^ | (Al-Bassam *et al.*, 2018) |
| SVJH8 | *rmdB*::*apr*; attBΦBT1::p3xFLAG; Apr^R^, Hyg^R^ | (Haist *et al.*, 2020) |
| SVBC1 | *rmdB*::*apr*, *attBΦBT1*::p3xFLAG-*rmdB^ΔTM^* Apr^R^, Hyg^R^ | This Work |
| SVBC2 | *rmdB*::*apr*, *attBΦBT1*::p3xFLAG-*rmdB^ΔGGDEF^* Apr^R^, Hyg^R^ | This Work |
| SVBC4 | *rmdB*::*apr*, *attBΦBT1*::p3xFLAG-*rmdB^AAA^* Apr^R^, Hyg^R^ | This Work |
| ***E. coli*** | | |
| DH5α | *fhuA2 lac(del)U169 phoA glnV44 Φ80' lacZ(del)M15 gyrA96 recA1 relA1 endA1 thi-1 hsdR17* | Thermo Fisher Scientific |
| ET12567/pUZ8002 | *dam*, *dcm*, *hsd*; Kan^R^, Cm^R^ | (Paget *et al.*, 1999) [1] |
| BTH101 | F^-^, *cya-99, araD139, galE15, galK16, rpsL1 (Str ^r^), hsdR2, mcrA1, mcrB1* | Euromedex |
| BL21 (DE3) pLysS | F– *ompT hsdS*(rB– mB–) *gal dcm* λ(DE3), Cm^R^ | Promega |
| BL21 (DE3) pLysS Rosetta^TM^ | F- *ompT hsdSB*(rB- mB-) *gal dcm* (DE3) pLysSRARE (Cam^R^) | Novagene |
| **Plasmids / NT plasmid collection or strain collection number** | | |
| pIJ10914 | pCOLADuet-1 with *His-whiG* (*vnz_26215*) in MCS1 and *rsiG (vnz_19430)* in MCS2, Kan^R^ | (Gallagher *et al.*, 2020) |
| pECJH14 | pGEX-6p1-*rmdB* (*vnz_25525*) (GST-tagged) | (Haist *et al.*, 2020) |
|  | pET15b/GST-RmdBGGDEF | This work |
| pUZ8002 | RP4 derivative with defective oriT; Kan^R^ | (Paget *et al.*, 1999) [1] |
| p3xFLAG | pIJ10770 derivative containing *3xFLAG* sequence downstream of MCS; Hyg^R^ | (Al-Bassam *et al.*, 2018) |
| pSVJH02 | p3xFLAG-*rmdB*, native promoter, Hyg^R^ | (Al-Bassam *et al.*, 2018) |
| pSVBC1 | p3xFLAG-*rmdB^ΔTM^*, native promoter, Hyg^R^ | This work |
| pSVBC2 | p3xFLAG-*rmdB^ΔGGDEF^*, native promoter, Hyg^R^ | This work |
| pSVBC4 | p3xFLAG-*rmdB^AAA^*, native promoter, Hyg^R^ | This work |
| pUT18C-zip | ori ColE1, T18-zip, Amp^R^ | (Karimova *et al.*, 1998) |
| pKT25-zip | ori p15A, T25-zip, Kan^R^ | (Karimova *et al.*, 1998) |
| pUT18C | ori ColE1, T18 fragment N-terminal, Amp^R^ | (Karimova *et al.*, 1998) |
| pUT18 | ori ColE1, T18 fragment C-terminal, Amp^R^ | (Karimova *et al.*, 1998) |
| pKT25 | ori p15A, T25 fragment N-terminal, Kan^R^ | (Karimova *et al.*, 1998) |
| pKNT25 | ori p15A, T25 fragment C-terminal, Kan^R^ | (Karimova *et al.*, 1998) |
| pECBC9 | pKT25_*rmdB*^ΔTM(Δ1-238aa)^, Kan^R^ | This work |
| pECBC10 | pUT18C_*rmdB*^ΔTM(Δ1-238aa)^, Amp^R^ | This work |
| pNT470 | pUT18-*cdgA*, Amp^R^ | This work |
| pNT471 | pKT25-*cdgA*, Kan^R^ | This work |
| pNT472 | pUT18-*cdgB*, Amp^R^ | This work |
| pNT473 | pKT25-*cdgB*, Kan^R^ | This work |
| pNT474 | pUT18-*cdgC*, Amp^R^ | This work |
| pNT475 | pKT25-*cdgC*, Kan^R^ | This work |
| pNT476 | pUT18-*cdgD*, Amp^R^ | This work |
| pNT477 | pKT25-*cdgD*, Kan^R^ | This work |
| pNT478 | pUT18-*cdgE*, Amp^R^ | This work |
| pNT479 | pKT25-*cdgE*, Kan^R^ | This work |
| pNT446 | pKNT25-*cdgF*, Kan^R^ | This work |
| pNT480 (pECJH5) | pUT18-*rmdA*, Amp^R^ | This work |
| pNT481 (pECJH3) | pKT25-*rmdA*, Amp^R^ | This work |
| pNT482 | pUT18-*hdgA*, Amp^R^ | This work |
| pNT483 | pKT25-*hdgA*, Kan^R^ | This work |
| pNT484 | pUT18-*hdgB*, Amp^R^ | This work |
| pNT485 | pKT25-*hdgB*, Kan^R^ | This work |
|  | pUT18C-*rsiG*, Amp^R^ | K. Gallagher & M. Buttner, JIC |
| pIJ10911 | pKT25-*rsiG*, Kan^R^ | Gallagher et al, 2020 |
| pIJ10909 | pUT18-*whiG*, Amp^R^ | Gallagher et al, 2020 |
| pIJ10907 | pKT25-*whiG*, Kan^R^ | Gallagher et al, 2020 |
| **Oligonucleotides used for generation of mutagenized *rmdB* variants in p3xFLAG** | | |
| **Name** | **Sequence 5´-3´** | **Purpose** |
| PRBC3_FR | TCCACCCTGTGGATCGCCCG | To delete the TM region (1-243 aa) from p3xFLAG-*rmdB* to construct p3xFLAG-*rmdB^ΔTM^* using Site-directed Mutagenesis |
| PRBC6_RV | CATTTCCGTCCCTCTCACAGG | To delete the TM region (1-243 aa) from p3xFLAG-*rmdB* to construct p3xFLAG-*rmdB^ΔTM^* using Site-directed Mutagenesis |
| GGDEF_LF_Fr | CAAGCTTAGATTCTCTCATAtgctcccggccccctcgcga | To delete the GGDEF region (258-421 aa) from p3xFLAG-*rmdB* to construct p3xFLAG-*rmdB^ΔGGDEF^* using Gibson Assembly |
| GGDEF_LF_Rv | AGCCGGTCGGGGGTGTTGGAgagctgttcctcggcgcggg | To delete the GGDEF region (258-421 aa) from p3xFLAG-*rmdB* to construct p3xFLAG-*rmdB^ΔGGDEF^* using Gibson Assembly |
| GGDEF_RF_Fr | CCCGCGCCGAGGAACAGCTCtccaacacccccgaccggct | To delete the GGDEF region (258-421 aa) from p3xFLAG-*rmdB* to construct p3xFLAG-*rmdB^ΔGGDEF^* using Gibson Assembly |
| GGDEF_RF_Rv | CTCGAGAGATGTACACCTAGgctggacgacctggccggag | To delete the GGDEF region (258-421 aa) from p3xFLAG-*rmdB* to construct p3xFLAG-*rmdB^ΔGGDEF^* using Gibson Assembly |
| AAA_LF_Fr | caagcttagattctctcatatgCTCCCGGCCCCCTCGCGA | For EAL to AAA exchange of p3xFLAG-*rmdB* to construct p3xFLAG-*rmdB^AAA^* using Gibson Assembly |
| AAA_LF_Rv | GCGGACGGCCGCGGCGAGGCCGGCGACCTGGCCG TCGAAC | For EAL to AAA exchange of p3xFLAG-*rmdB* to construct p3xFLAG-*rmdB^AAA^* using Gibson Assembly |
| AAA_RF_Fr | GTTCGACGGCCAGGTCGCCGGCCTCGCCGCGGCC GTCCGC | For EAL to AAA exchange of p3xFLAG-*rmdB* to construct p3xFLAG-*rmdB^AAA^* using Gibson Assembly |
| AAA_RF_Rv | ctcgagagatgtacacctaggCTGGACGACCTGGCCGGAGGGG | For EAL to AAA exchange of p3xFLAG-*rmdB* to construct p3xFLAG-*rmdB^AAA^* using Gibson Assembly |
| **Oligonucleotides used for cloning of *rmdB* into pGEX** | | |
| PRJH28 | GCTGggatccggCTCCACCCTGTGGATCGCCC | To construct pGEX-*rmdB* |
| PRJH29 | CATGcccgggCTACTGGACGACCTGGCCGG | To construct pGEX-*rmdB* |
| **Oligonucleotides used for cloning of the GGDEF domain of RmdB into pET15b** | | |
| 15b_GSTGGDEF_Nco_Fwd | gatataccatgggcTCCCCTATACTAGGTTATTG | To construct pET15b/GSTRmdBGGDEF |
| 15b_GSTGGDEF_Bgl_Rev | GAGTCAagatctCTAGGTGTTGGAGTCGCGCTTG | To construct pET15b/GSTRmdBGGDEF |
| **Oligonucleotides used for cloning of genes encoding GGDEF-, EAL- and HD-GYP domain proteins into BACTH vectors** | | |
| BTH_XbaI_cdgA_fw | cagTCTAGAgAGCGGAACCCCCGAAGAAC | for pNT470 (pUT18-*cdgA*) and pNT471 (pKT25-*cdgA*) |
| BTH_KpnI_cdgA_rv | cagGGTACCcgGACCCGCAGCGGTTCCG |  |
| BTH_XbaI_cdgB_fw | cagTCTAGAgGAGACCGAGTCGGAGC | for pNT472 (pUT18-*cdgB*) and pNT473 (pKT25-*cdgB*) |
| BTH_KpnI_cdgB_rv | cagGGTACCcgCCGCCGGTGCACCTTG |  |
| BTH_XbaI_cdgC_fw | GACCtctagaGGTGCCGGACCAGGCC | for pNT474 (pUT18-*cdgC*) |
| BTH_KpnI_cdgC-st_rv | GACggtaccCGAGTGGGTGGGACAGGCG |  |
| BTH_XbaI_cdgC_fw | GACCtctagaGGTGCCGGACCAGGCC | for pNT475 (pKT25-*cdgC*) |
| BTH_KpnI_cdgC _rv | GACggtaccTCAAGTGGGTGGGACAGGC |  |
| BTH_XbaI_cdgD_fw | cagTCTAGAgGACGCGCAGACGATCGC | for pNT476 (pUT18-*cdgD*) and pNT477 (pKT25-*cdgD*) |
| BTH_KpnI_cdgD_rv | cagGGTACCcgGTGTGTGTTGCGCCTGCC |  |
| BTH_XbaI_cdgE_fw | cagTCTAGAgGGTGAGGACGTACGGCTG | for pNT478 (pUT18-*cdgE*) and pNT479 (pKT25-*cdgE*) |
| BTH_KpnI_cdgE_rv | cagGGTACCcgCCCGTCCCCGCCCTC |  |
| BTH_HindIII_cdgF_fw | TCAGCCAAGCTTAATGTCGCTGCGCCAGC | for pNT446 (pKNT25-*cdgF*) |
| BTH_EcoRI_cdgF_rev | GTCACTGAATTCACCGGCTCCGGCGTCCGG |  |
| PRJH3 | ATAaagcttgGTGAAGGTCCCGTCGCAG | for pNT480 (pUT18-*rmdA*) |
| PRJH4 | ATAgaattcgaGACGGCGTCCGCCAG |  |
| PRJH1 | ATAtctagagGTGAAGGTCCCGTCGCAG | for pNT481 (pKT25-*rmdA*) |
| PRJH2 | ATAgaattcTCAGACGGCGTCCGCCAG |  |
| BTH_XbaI_hdgA_fw | cagTCTAGAgGTGCGAATCCGCTCC | for pNT482 (pUT18-*hdgA*) and pNT483 (pKT25-*hdgA*) |
| BTH_KpnI_hdgA_rv | cagGGTACCcgTGCGCGTTCCCTGGG |  |
| BTH_XbaI_hdgB_fw | cagTCTAGAgAGACCCGGCGCCCTCAC | for pNT484 (pUT18-*hdgB*) and pNT485 (pKT25-*hdgB*) |
| BTH_KpnI_hdgB_rv | cagGGTACCcgAGCGGGGGCGGAGGTC |  |
| PRBC15_Fr | CTCATCGCCCTCGACTCCAC | For deletion of the TM region (1-238 aa) from pKT25-*rmdB* and pUT18C-*rmdB* to construct pECBC9 (pKT25-*rmdB^ΔTM^)* and pECBC10 (pUT18C-*rmdB^ΔTM^)* using Site-directed Mutagenesis |
| PRBC15_Rv | ctctagAGTCGACCCTGCAG | For deletion of the TM region (1-238 aa) from pKT25-*rmdB* to construct pECBC9 (pKT25-*rmdB^ΔTM^)* using Site-directed Mutagenesis |
| PRBC16_Rv | ctctagAGTCGACCTGCAG | For deletion of the TM region (1-238 aa) from pUT18C-*rmdB* to construct pECBC10 (pKT25-*rmdB^ΔTM^)* using Site-directed Mutagenesis |

Reference

1. Paget, M.S., Chamberlin, L., Atrih, A., Foster, S.J., and Buttner, M.J. (1999). Evidence that the extracytoplasmic function sigma factor sigmaE is required for normal cell wall structure in *Streptomyces coelicolor* A3(2). *J Bacteriol* 181, 204-211.
