# Supplementary material for: Dual-function enzyme acts as a global c-di-GMP sink and local anti sigma factor antagonist to drive cellular differentiation
Source: PLoS Genet. 2026 Jun 3;22(6):e1012161. doi: 10.1371/journal.pgen.1012161 (PMC13232838; doi:10.1371/journal.pgen.1012161)
Supplement: Attachment — Submitted filename: PLOS Genetics_Response to Reviewers_Cinar Cakmak.pdf [file pgen.1012161.s011.pdf]

ifmb Institut für Mikrobiologie

Leibniz Universität Hannover

Gottfried Wilhelm Leibniz Universität Hannover,
Institut für Mikrobiologie, Herrenhäuser Straße 2, D-30419 Hannover

Editor
PLOS Genetics

Naturwissenschaftliche Fakultät

Institut für Mikrobiologie

Prof. Dr. Natalia Tschowri
Tel. +49 511 762 5241
Fax +49 511 762 5287
E-Mail: tschowri
@ifmb.uni-hannover.de

30-Apr-26

Dear Editor,

Thank you very much for serving as our editor and providing helpful comments from the reviewers. We have carefully addressed each of the reviewer's critiques (see in blue below).

Response to Reviewers
Reviewer #1:

This manuscript by Çakmak et al., advances our understanding of how c-di-GMP signalling is terminated to allow developmental progression towards sporulation in Streptomyces venezuelae. The authors identify RmdB as a dual-function phosphodiesterase that integrates global degradation of c-di-GMP with local, protein–protein interactions that directly impact sigma/anti-sigma regulation. These findings are novel and of broad interest to the bacterial signalling and Streptomyces development communities. The manuscript is clearly written, logically structured and easy to follow. The experimental strategy is well thought out and combines genetics, biochemical assays, protein-protein interaction analyses and phenotyping to back up the manuscript's conclusions. The use of co-immunoprecipitation, co-elution, and bacterial two-hybrid assays provides complementary and mutually reinforcing support for the proposed model. The identification of RmdB as an interaction hub connecting multiple DGCs further strengthens the idea that spatial and local control of c-di-GMP signalling is central to developmental regulation in Streptomyces venezuelae. Overall, this is a high-quality study that makes a significant conceptual contribution to the field of signal transduction and developmental biology in Streptomyces venezuelae. I have no concerns or suggestions regarding the experimental design, data quality or interpretation. My only suggestions would be to briefly clarify in the discussion how conserved RmdB is within Streptomyces species and whether other phosphodiesterase-sigma factor interactions have been characterised in any other

Besucheradresse:
Herrenhäuser Straße
D-30419 Hannover
www.ifmb.uni-hannover.de

Sekretariat:
Tel. +49 511 762 4359
Fax +49 511 762 5287

www.uni-hannover.de

organisms, and if so, how do these compare to the RmdB-WhiG interaction studied here. These minor suggestions do not detract from the strength of the work presented here.

We are very thankful for this very positive feedback. On page 7, we state "RmdB belongs to the most conserved c-di-GMP turnover enzymes in the *Streptomyces* pan genome and it is the most highly expressed of the c-di-GMP genes in S. venezuelae (Al-Bassam et al., 2018)." On page 12, we highlight "Our findings support a model in which RmdB functions as an anti sigma factor antagonist that competes with the anti sigma factor RsiG for binding to its partner protein σ^{WhiG} - a previously unrecognized role for a phosphodiesterase in bacteria."

Reviewer #2:

In this study, Çakmak et al explored the role of the Streptomyces phosphodiesterase RmdB in lowering the level of c-di-GMP within cells to allow development to progress. RmdB is a diguanylate cyclase/phosphodiesterase hybrid protein that contains the GGDEF and EAL domains, as well as an additional N-terminal transmembrane domain that anchors the protein to the membrane. The EAL and GGDEF domains are both required for RmdB to function in the timing of developmental progression. Based on the phenotype, the authors performed co-immunoprecipitation and in vivo protein-protein interaction studies using bacterial two-hybrid (BACTH) analysis. These studies revealed that the RmdB GGDEF domain interacts directly with the sigma factor WhiG, thereby assisting in the release from its anti-sigma factor, RsiG. Furthermore, additional BACTH analyses suggest that RmdB interacts with other DGCs, supporting a model in which RmdB acts as a hub that coordinates local interactions and likely the activity of other enzymes involved in c-di-GMP synthesis and degradation.

Overall, this is a well-conducted study with a clear, easy-to-follow set of experiments. The results generally justify the conclusions (see below), though the summary model is preliminary. I believe the interaction studies, but I find it somewhat unsatisfactory that there are so many obvious experiments that the authors are probably planning to perform in the future are not included. How does the GGDEF domain interact with the sigma/anti-sigma complex? Does the interaction affect RmdB activity? Does RmdB binding loosen the interaction between sigma and anti-sigma factors? How does RmdB interact with the other enzymes involved in c-di-GMP synthesis and degradation, and how does this interaction affect their activity? Is there sufficient RmdB to allow binding of all the potential clients? The last paragraph of the Results section is essentially one big question. Given the wealth of data that can be gained from this system, I wonder if this manuscript is a bit premature – but that is not for me to judge.

We are very thankful for careful review of our manuscript and agree that discoveries presented here raise many exciting follow-up questions. However, addressing all of them goes beyond the scope of this manuscript.

Questions/remarks

Abstract, line 25 and elsewhere: rather a question – is it common to refer to an anti-sigma factor as ‚anti sigma'?

In the reference paper (Gallagher et al 2020, Mol Cell), the authors use the term "anti-sigma" for RsiG: "c-di-GMP Arms an Anti-sigma to Control Progression of Multicellular Differentiation in Streptomyces". However, we agree that it is more precise to use anti sigma factor. We have changed anti-sigma to anti sigma factor in the entire the text.

Introduction: Does the RmdB transmembrane region have a function of its own, e.g., in signal perception?

Excellent question! Unfortunately, we have no answer yet. We cannot identify a specific sensory region/motif using prediction tools.

84, just as a suggestion: The authors may also mention the compartimentalization of c-di-GMP signalling in Caulobacter, where (as in Streptomyces) the cell cycle progression is regulated by this second messenger.

Thank you very much for this suggestion. True, Caulobacter is also a great system for illustrating the local signalling concept in c-di-GMP signalling. However, it also involves many different players and components that would require good explanation. Thus, on page 4, we have added a key review from the Jenal group so that the reader can find all the needed information if interested.

116, end of paragraph, what about RmdA?

Thank you for spotting! We have now added on page 6: "RmdA is an active PDE with residual DGC activity (Haist et al., 2020)"

134, although the difference appears to be clear for most of the data, some statistics would truly demonstrate the significance.

We have included a new version of Figure 1B, showing statistical analysis using a 2-way ANOVA test.

Figure 3B/C show some cropped data. It would be good, if the non-processed western blots could be added to the supplementary data.

Agree. We have added a non-processed version of the image shown in Figure 3B in Supplementary Figure S2 and Supplementary Figure S3A+B showing non-processed versions of Westernblot images presented in Figures 3 C and D.

169, Are the authors suggesting that the GGDEF domain binds to the WhiG-RsiG complex via bound c-di-GMP? Does AlphaFold's structure/interaction prediction suggest this? What would a prediction involving the individual proteins (WhiG with RmdB-GGDEF and RsiG with RmdB-GGDEF) look like? Does this support the

hypothesis? Does the analysis suggest any critical interaction surfaces or residues that could be substituted?

We thank the reviewer for raising this point. Our data do not allow us to conclude that RmdB binds the WhiG-RsiG complex specifically through direct recognition of the c-di-GMP dimer bound within the complex. Rather, the exposed position of the intercalated c-di-GMP in the published RsiG-(c-di-GMP)$_2$-WhiG structure prompted us to test whether RmdB might be involved in the disassembly of the complex.

Following your suggestion, we used AlphaFold3 to model complexes of full-length RmdB and of the isolated GGDEF domain with WhiG-RsiG, WhiG alone, and RsiG alone. However, all predictions yielded low interface confidence (iPTM ~0.2–0.4), which is below the range that would justify interpretation of a specific docking mode or assignment of contact residues. We therefore consider these models inconclusive. We also note that such predictions are particularly challenging for multiprotein/multidomain and membrane-associated proteins such as RmdB, whose transmembrane region contributes to protein stability, and for which the physiologically relevant oligomeric state is not yet fully resolved.

For this reason, our mechanistic interpretation is based primarily on the experimental evidence rather than on the structural predictions. Specifically, we show that deletion of the GGDEF domain strongly reduces co-immunoprecipitation of WhiG (Figure 3D), whereas the isolated GGDEF domain is sufficient to co-purify with WhiG (Supplementary Figure 6). Together, these data support a model in which the GGDEF domain of RmdB interacts with WhiG directly and thereby disfavors or competes with RsiG binding, but they do not yet establish whether bound c-di-GMP makes a direct contribution to this interface.

204, please provide a control with non-His-tagged WhiG

We thank the reviewer for pointing out the importance of this control. We agree that it is essential to exclude nonspecific binding of the RmdB$_{GGDEF}$ construct to the Ni-NTA matrix in the absence of His-WhiG. We have now added this control experiment and present it in the modified Suppplementary Figure 6. In the revised analysis, GST-RmdB$_{GGDEF}$ was subjected to Ni-NTA purification in the presence or absence of His-WhiG. GST-RmdB$_{GGDEF}$ was recovered from the Ni-NTA matrix only when His-WhiG was co-expressed, but not in its absence. This control therefore confirms that the GGDEF domain does not bind to the matrix on its own.

For this control, we used a GST fusion rather than the MBP fusion shown previously in the supplement. We chose GST for two reasons. First, GST-tagged RmdB constructs were used throughout the main co-purification experiments in the manuscript, so using GST here provides a directly comparable and internally consistent format. Second, repeating the experiment with a different fusion tag strengthens the conclusion by showing that co-purification of the GGDEF domain with His-WhiG is not a specific property of the MBP tag. We therefore consider the new GST-based control to be a more stringent demonstration that the GGDEF domain of RmdB specifically interacts with WhiG.

223, how was the stability tested?

Stability of full size RmdB carrying a C-terminal T18 fragment was tested using Westernblot analysis and the CyaA (3D1) antibody against the T18 fragment. Representative results are shown here. While we could not detect a signal for RmdB-T18, a specific band of the expected size (72 kDa) is visible for RmdB$^{\Delta TM}$-T18 in the last lane.

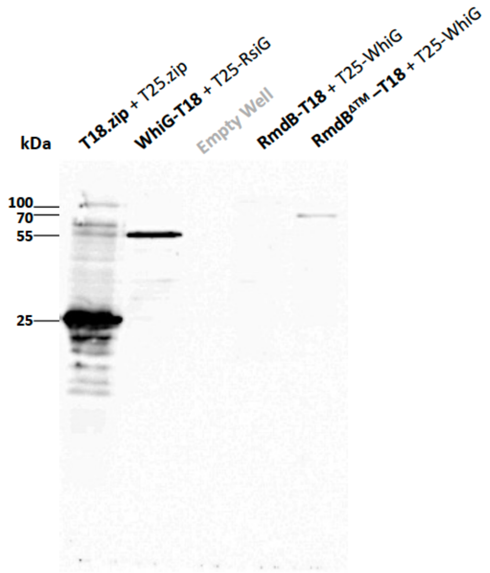

Figure: Western analysis of samples of BTH101 strains carrying listed BTH plasmid combinations. The BTH101 strains co-expressing listed plasmids were grown in LB supplemented with 0.5 mM IPTG, ampicillin (100 µg/µl) and kanamycin (50 µg/µl) at 37°C, 180 rpm, overnight. An equal amount of total protein (20 µg) was loaded to each lane. Westernblot analysis was performed using the Cya A (3D1) antibody against the T18 fragment. Expected band sizes: T18.zip: 26 kDa, WhiG-T18: 52 kDa, RmdB-T18: 97 kDa, RmdB$^{\Delta TM}$ –T18: 72 kDa.

Reviewer #3:

Cakmak et al. investigated the role of the phosphodiesterase RmdB in c-di-GMP signaling and cell fate decisions in Streptomyces. They report two main findings: (i) Consistent with their previous study (Haist et al., Mol Microbiol, 2020), the authors demonstrate that RmdB functions as a phosphodiesterase. Accordingly, global c-di-GMP levels are elevated in the rmdB mutant, which correlates with delayed development. Both the EAL domain (essential for phosphodiesterase activity) and the GGDEF domain (typically a diguanylate cyclase) are required for RmdB function in vivo. While this part of the study is technically sound and straightforward, its novelty and mechanistic insight are limited.
(ii) To explore the potential alternative function of the GGDEF domain, the authors propose that RmdB acts as a hub for protein-protein interactions. Pulldown experiments suggest an interaction between RmdB and the sigma factor WhiG, leading to the hypothesis that this interaction antagonizes binding of the anti-sigma factor RsiG to the sigma factor. Unfortunately, the interaction

was not supported by the bacterial two-hybrid system. Instead, this assay revealed interactions between RmdB and four diguanylate cyclases. Overall, this second line of research is less well developed, and the available experimental evidence is not yet sufficiently strong enough to substantiate the proposed, appealing model.

We are very thankful for careful review of our manuscript and your feedback.

Questions and comments:
Determining protein-protein interactions using a single experimental approach carries inherent risks, particularly when a complementary approach (in this case, the BACTH assay) does not support the initial findings. The following questions and suggestions are intended to strengthen confidence in the reported results.

Thank you very much for sharing your view on this part of the experiments. We would like to highlight, that two independent experimental set-ups demonstrate interaction between RmdB and WhiG. One is based on co-expression and co-elution of RmdB and WhiG using *E. coli* as a host (data shown in Figure 3B) The second approach is based on co-immunoprecipitation using the native host, i.e. *S. venezuelae* for expression and co-elution (Figure 3D). We do agree that it is somewhat disappointing that BTH assays do not confirm the observed interactions using co-expression and co-elution in either *E. coli* or *S. venezuelae*, however, we emphasize methodological limitations of BHT assays in the discussion section.

1. Figure 3 lacks information on reproducibility. Does it show one representative example from multiple independent experiments?

Good point, thank you! In the legend to Figure 3, we have now added the information that data shown in Figure 3 are representative images of three independent experiments.

2. Figure 3B: The interaction between RmdB and the sigma factor could be validated by a reciprocal pulldown using Glutathione Sepharose.

Thank you for this great suggestion! We have performed the suggested experiment and confirmed that His-WhiG binds to glutathione Sepharose when co-expressed with GST-RmdB, but not when expressed with RsiG only. These data further confirm that RmdB and WhiG interact and are now shown in the new Supplementary Figure S5.

3. Figure 3B: Given that WhiG-specific antibodies are available (Figure 3D), an informative follow-up experiment would be to recombinantly produce His-tagged RsiG together with native WhiG to test the proposed partner-switching model.

We thank the reviewer for this valuable suggestion. We have performed the proposed follow-up experiment by co-expressing His-tagged RsiG together with

untagged WhiG in the presence or absence of GST-RmdB, followed by purification on Ni-NTA and detection of co-purified WhiG by western blotting (Figure below). In this assay, substantially less WhiG co-purified with His-RsiG when RmdB was co-expressed, while His-RsiG itself was efficiently recovered in both conditions. Quantification of the WhiG/His-RsiG signal ratio showed a marked reduction in the presence of RmdB, from ~0.85–0.90 without RmdB to ~0.29–0.37 with RmdB. These data support the conclusion that RmdB interferes with formation and/or stability of the WhiG–RsiG complex and are therefore consistent with the proposed partner-switching model. At the same time, we note that the partner-switching model is already supported by several independent lines of evidence presented in the manuscript. First, in the original experiment shown in Fig. 3B, co-expression of RmdB with His-WhiG and RsiG reduces the amount of RsiG recovered together with His-WhiG. Second, co-immunoprecipitation experiments in *S. venezuelae* show that RmdB efficiently associates with WhiG in vivo (Fig. 3C-D), whereas RsiG is not detected in the eluates under these conditions (Fig. S4). Taken together, these observations already support a model in which RmdB interacts with WhiG and disfavors its association with RsiG. We therefore consider the new experiment confirmatory rather than necessary for establishing the main conclusion and did not include these data in the revised manuscript.

A. **B.**

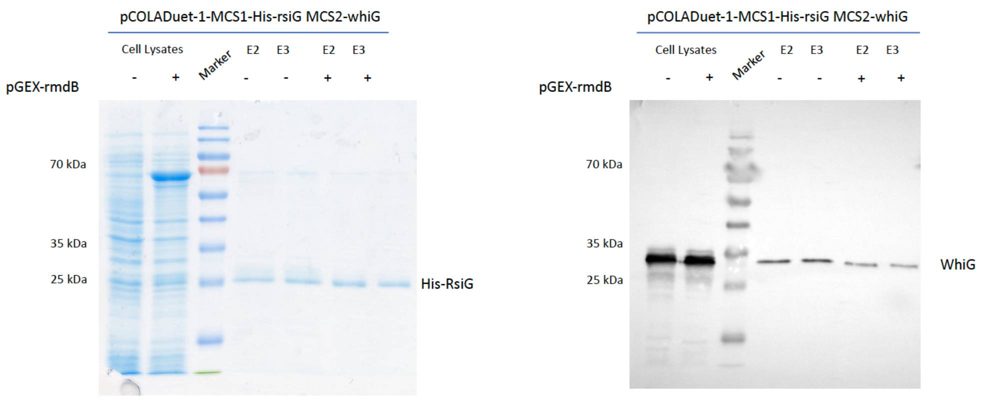

C.

Quantification of Co-purification of His-RsiG and WhiG with and without RmdB

Sample	Intensity His-RsiG (AUC)	Intensity WhiG (AUC)	Ratio WhiG/His-RsiG
E2_pCOLADuet-1-MCS1-His-rsiG MCS2-whiG	28209,688	25461,442	0,902577937
E3_pCOLADuet-1-MCS1-His-rsiG MCS2-whiG	30211,638	25802,028	0,854042671
E2_pCOLADuet-1-MCS1-His-rsiG MCS2-whiG + pGEX-rmdB	38151,032	13962,35	0,365975683
E3_pCOLADuet-1-MCS1-His-rsiG MCS2-whiG + pGEX-rmdB	33189,668	9465,551	0,285195712

Figure: Co-purification of His-RsiG from *E. coli* BL21 pLysS Rosetta using Ni-NTA-agarose matrix. His-*rsiG* and tag less *whiG* were expressed from pCOLADuet-1 from two separate multiple cloning sites. GST-tagged *rmdB* was expressed from the pGEX.6p1 vector. Gene expression was induced with 250 µM IPTG and cells were incubated in LB at 16°C overnight. Purification of His-RsiG was performed using Ni-NTA-agarose matrix. **A.** Cell lysates and the eluates were analysed via

SDS-PAGE. **B.** Cell lysates and eluates were analysed using western blotting and the polyclonal anti-WhiG antibodies (Gallagher et al 2020, Mol Cell). Equal protein amounts in the eluate fractions were loaded on the gels. **C.** Quantification was performed using ImageJ 1.54p.

4. Figure 3B: Can the pulldown experiments be repeated using purified proteins? GST- and His-tagged proteins should be easy to obtain and would confirm at least the RmdB-sigma factor interaction.

We thank the reviewer for this constructive suggestion. We agree that a pulldown experiment using separately purified components would provide an additional line of support for the RmdB–σ^{WhiG} interaction. During the revision, we therefore invested substantial effort into obtaining purified σ^{WhiG} for such experiments. However, despite repeated attempts and careful optimization trials, we were not able to purify σ^{WhiG} in a sufficiently stable and soluble form, and therefore could not perform a binding assay with individually purified proteins within the revision period, that we even extended due to these reasons.

Importantly, our conclusion does not rely on the co-expression/co-purification result shown in Fig. 3B alone. The interaction is supported by multiple independent observations: (i) co-elution of RmdB with His-σ^{WhiG} after co-expression in *E. coli* (Fig. 3B), (ii) reciprocal recovery of His-σ^{WhiG} on glutathione resin in the presence of GST-RmdB (new Fig. S5), (iii) co-immunoprecipitation of σ^{WhiG} with FLAG-tagged RmdB from *S. venezuelae* (Fig. 3C), and (iv) the strong reduction in σ^{WhiG} recovery when the GGDEF domain is deleted (Fig. 3D), together with co-purification of the isolated GGDEF domain with His-σ^{WhiG} (Fig. S6). Taken together, these multiple data strongly support a specific interaction between RmdB and σ^{WhiG}, even though direct reconstitution from individually purified proteins could not be achieved at this stage.

5. Figure 3C, D: While it is reassuring that the GGDEF variant is present in similar amounts, it remains possible that the protein, which lacks more than 150 amino acids, is not properly folded and therefore unable to engage in proper interactions. Does AlphaFold predict a structured protein or this variant?

We thank the reviewer for this important point. We agree that, in principle, deletion of the GGDEF domain could affect folding of the remaining protein. To address this, we performed AlphaFold3 predictions for the RmdBΔGGDEF variant. These models predict that the truncated protein remains structured, with the transmembrane region and the EAL domain adopting an overall architecture highly similar to that of full-length RmdB. Thus, the in silico analysis does not support gross misfolding or collapse of the remaining domains in the absence of the GGDEF region. We have included these structural predictions as a new Supplementary Figure 7 and added this information in the text.

6. Can AlphaFold modeling be used to support the proposed RmdB interaction partners?

Thank you very much for this question/suggestion, which was also raised by reviewer#2 (see above). We used AlphaFold3 to model complexes of full-length RmdB and of the isolated GGDEF domain with WhiG-RsiG, WhiG alone, and RsiG alone. However, all predictions yielded low interface confidence (iPTM ~0.2–0.4), which is below the range that would justify interpretation of a specific docking mode or assignment of contact residues. We therefore consider these models inconclusive. We also note that such predictions are particularly challenging for multiprotein/multidomain and membrane-associated proteins such as RmdB, whose transmembrane region contributes to protein stability, and for which the physiologically relevant oligomeric state is not yet fully resolved.

7. The BACTH experiments were performed using the cytosolic version of RmdB. If the extensive transmembrane region regulates the interactome of the proposed hub protein, it may be worthwhile to fuse the T18 or T25 to the C-terminus of the full-length protein and reassess interactions.

Thank you very much for raising this concern. Initially, we indeed started our analyses with full size RmdB carrying an T18/T25 fragment at the C-terminus. However, our westernblot analysis using the Cya A (3D1) antibody against the T18 fragment revealed that this protein version is instable, while RmdB$^{\Delta TM}$ –T18 was detectable. Please also see a representative image in our response to reviewer #2.
